# Supplementary material for: Navigating the complexities of the forest land sharing vs sparing logging dilemma: analytical insights through the governance theory of social-ecological systems dynamics
Source: PeerJ. 2024 Jan 29;12:e16809. doi: 10.7717/peerj.16809 (PMC10832625; doi:10.7717/peerj.16809)
Supplement: Supplemental Information 1 — The raw results from simulations used to construct the tables can be found in the supplementary spreadsheet. [file peerj-12-16809-s001.pdf]

Supplementary file S1 of the paper “Navigating the complexities of the forest land sharing vs sparing logging dilemma: analytical insights through the governance theory of social-ecological systems dynamics”

Jean-Baptiste Pichancourt[1]

[1]**Université Clermont-Auvergne (UCA), INRAE, Laboratoire d’Ingénieurie des Systèmes Complèxes (UR LISC), Centre de Clermont-Ferrand, 9 avenue Blaise Pascal, F-63178 Aubière, France**

Corresponding author:
Jean-Baptiste Pichancourt[1]

Email address: jean-baptiste.pichancourt@inrae.fr

ABSTRACT

This supplementary file presents the detailed values used to produce Table 2 when the criteria of comparison is the forest-level average above-ground carbon stock (Table S1) or the average annual timber harvested (Table S2. The raw results from simulations used to construct the tables can be found in the supplementary spreadsheet.

Mono-specific	Carbon stock	Reference for comparison: land-sharing forest with different number of species harvested								
stand planted*	$[tC.ha^{-1}]$	1 species	2 species	3 species	4 species	5 species	6 species	7 species	8 species	Sparlines
Condition to meet: at least the performance of land-sharing with no collective constraints on harvesting										
		166	158	152	146	140	132	125	117	
sp#1	34.23	0%	0%	0%	0%	0%	0%	0%	0%	
sp#2	15.7	0%	0%	0%	0%	0%	0%	0%	0%	
sp#3	149	0%	0%	0%	2%	6%	11%	16%	22%	
sp#4	30.7	0%	0%	0%	0%	0%	0%	0%	0%	
sp#5	51.7	0%	0%	0%	0%	0%	0%	0%	0%	
sp#6	16.4	0%	0%	0%	0%	0%	0%	0%	0%	
sp#7	111	0%	0%	0%	0%	0%	0%	0%	0%	
sp#8	67	0%	0%	0%	0%	0%	0%	0%	0%	
Condition to meet: at least the performance of land-sharing with compulsary harvest of sp#4										
		177	169	161	152	143	135	127	117	
sp#1	34.23	0%	0%	0%	0%	0%	0%	0%	0%	
sp#2	15.7	0%	0%	0%	0%	0%	0%	0%	0%	
sp#3	149	0%	0%	0%	2%	6%	11%	16%	22%	
sp#4	30.7	0%	0%	0%	0%	0%	0%	0%	0%	
sp#5	51.7	0%	0%	0%	0%	0%	0%	0%	0%	
sp#6	16.4	0%	0%	0%	0%	0%	0%	0%	0%	
sp#7	111	0%	0%	0%	0%	0%	0%	0%	0%	
sp#8	67	0%	0%	0%	0%	0%	0%	0%	0%	
Condition to meet: at least the performance of land-sharing with compulsary harvest of sp#3										
		83	97	106	112	115	116	116	117	
sp#1	34.23	0%	0%	0%	0%	0%	0%	0%	0%	
sp#2	15.7	0%	0%	0%	0%	0%	0%	0%	0%	
sp#3	149	44%	35%	29%	25%	23%	22%	22%	21%	
sp#4	30.7	0%	0%	0%	0%	0%	0%	0%	0%	
sp#5	51.7	0%	0%	0%	0%	0%	0%	0%	0%	
sp#6	16.4	0%	0%	0%	0%	0%	0%	0%	0%	
sp#7	111	25%	13%	5%	0%	0%	0%	0%	0%	
sp#8	67	0%	0%	0%	0%	0%	0%	0%	0%	
Condition to meet: at least the performance of land-sharing with forbidden harvest of sp#4										
		178	179	179	180	180	181	181	na	
sp#1	34.23	0%	0%	0%	0%	0%	0%	0%	na	
sp#2	15.7	0%	0%	0%	0%	0%	0%	0%	na	
sp#3	149	0%	0%	0%	0%	0%	0%	0%	na	
sp#4	30.7	0%	0%	0%	0%	0%	0%	0%	na	
sp#5	51.7	0%	0%	0%	0%	0%	0%	0%	na	
sp#6	16.4	0%	0%	0%	0%	0%	0%	0%	na	
sp#7	111	0%	0%	0%	0%	0%	0%	0%	na	
sp#8	67	0%	0%	0%	0%	0%	0%	0%	na	
Condition to meet: at least the performance of land-sharing with forbidden harvesting of sp#3										
		165	155	147	140	140	133	125	117	
sp#1	34.23	0%	0%	0%	0%	0%	0%	0%	0%	
sp#2	15.7	0%	0%	0%	0%	0%	0%	0%	0%	
sp#3	149	0%	0%	0%	1%	6%	11%	16%	21%	
sp#4	30.7	0%	0%	0%	0%	0%	0%	0%	0%	
sp#5	51.7	0%	0%	0%	0%	0%	0%	0%	0%	
sp#6	16.4	0%	0%	0%	0%	0%	0%	0%	0%	
sp#7	111	0%	0%	0%	0%	0%	0%	0%	0%	
sp#8	67	0%	0%	0%	0%	0%	0%	0%	0%	

Table S1. The percentage of land that can be spared for conservation varies depending on the type of mono-specific carbon plantation (using species sp#1 to sp#8). This was estimated by comparing the average above-ground forest level carbon stock over 100 years of simulations for each mono-specific stand and for land-shared forests described in ??. *The values of functional traits for species sp#1 to sp#8 are presented in ??. Simulation results and analysis to produce the table can be found in the supplementary file 1. The original model used to produce the results can be found in ?.

Mono-specific	Timber supply	Reference for comparison: land-sharing forest with different number of species harvested								
stand planted*	$[t.ha^{-1}.y^{-1}]$	1 species	2 species	3 species	4 species	5 species	6 species	7 species	8 species	Sparlines
Condition to meet: at least the performance of land-sharing with no collective constraints on harvesting										
		.49	1.51	2.46	2.46	3.69	4.01	4.95	5.6	
sp#1	2.79	83%	46%	12%	12%	0%	0%	0%	0%	
sp#2	.48	0%	0%	0%	0%	0%	0%	0%	0%	
sp#3	6.25	92%	76%	61%	61%	41%	36%	21%	10%	
sp#4	1.11	56%	0%	0%	0%	0%	0%	0%	0%	
sp#5	0.02	0%	0%	0%	0%	0%	0%	0%	0%	
sp#6	0.07	0%	0%	0%	0%	0%	0%	0%	0%	
sp#7	0.03	0%	0%	0%	0%	0%	0%	0%	0%	
sp#8	0.28	0%	0%	0%	0%	0%	0%	0%	0%	
Condition to meet: at least the performance of land-sharing with compulsary harvest of sp#4										
		.08	2.32	3.97	4.9	5.4	5.56	5.57	5.6	
sp#1	2.79	100%	14%	0%	0%	0%	0%	0%	0%	
sp#2	.48	98%	0%	0%	0%	0%	0%	0%	0%	
sp#3	6.25	100%	62%	36%	22%	14%	11%	11%	10%	
sp#4	1.11	99%	0%	0%	0%	0%	0%	0%	0%	
sp#5	0.02	67%	0%	0%	0%	0%	0%	0%	0%	
sp#6	0.07	89%	0%	0%	0%	0%	0%	0%	0%	
sp#7	0.03	74%	0%	0%	0%	0%	0%	0%	0%	
sp#8	0.28	97%	0%	0%	0%	0%	0%	0%	0%	
Condition to meet: at least the performance of land-sharing with compulsary harvest of sp#3										
		.54	1.27	1.99	2.71	3.43	4.14	4.86	5.6	
sp#1	2.79	80%	55%	29%	3%	0%	0%	0%	0%	
sp#2	.48	0%	0%	0%	0%	0%	0%	0%	0%	
sp#3	6.25	91%	80%	68%	57%	45%	34%	22%	10%	
sp#4	1.11	51%	0%	0%	0%	0%	0%	0%	0%	
sp#5	0.02	0%	0%	0%	0%	0%	0%	0%	0%	
sp#6	0.07	0%	0%	0%	0%	0%	0%	0%	0%	
sp#7	0.03	0%	0%	0%	0%	0%	0%	0%	0%	
sp#8	0.28	0%	0%	0%	0%	0%	0%	0%	0%	
Condition to meet: at least the performance of land-sharing with forbidden harvest of sp#4										
		.56	.56	.57	.58	.59	.59	.59	na	
sp#1	2.79	80%	80%	79%	79%	79%	79%	79%	na	
sp#2	.48	0%	0%	0%	0%	0%	0%	0%	na	
sp#3	6.25	91%	91%	91%	91%	91%	91%	91%	na	
sp#4	1.11	50%	49%	48%	48%	47%	47%	46%	na	
sp#5	0.02	0%	0%	0%	0%	0%	0%	0%	na	
sp#6	0.07	0%	0%	0%	0%	0%	0%	0%	na	
sp#7	0.03	0%	0%	0%	0%	0%	0%	0%	na	
sp#8	0.28	0%	0%	0%	0%	0%	0%	0%	na	
Condition to meet: at least the performance of land-sharing with forbidden harvesting of sp#3										
		.48	.94	1.76	2.79	3.87	4.85	5.55	na	
sp#1	2.79	83%	66%	37%	0%	0%	0%	0%	na	
sp#2	.48	0%	0%	0%	0%	0%	0%	0%	na	
sp#3	6.25	92%	85%	72%	55%	38%	22%	11%	na	
sp#4	1.11	57%	15%	0%	0%	0%	0%	0%	na	
sp#5	0.02	0%	0%	0%	0%	0%	0%	0%	na	
sp#6	0.07	0%	0%	0%	0%	0%	0%	0%	na	
sp#7	0.03	0%	0%	0%	0%	0%	0%	0%	na	
sp#8	0.28	0%	0%	0%	0%	0%	0%	0%	na	

Table S2. The percentage of land that can be spared for conservation varies depending on the type of mono-specific timber plantation (using species sp#1 to sp#8). This was estimated by comparing the average performances in timber supply over 100 years of simulations for each mono-specific stand and for land-shared forests described in ??. *The values of functional traits for species sp#1 to sp#8 are presented in ??. Simulation results and analysis to produce the table can be found in the supplementary file 1. The original model used to produce the results can be found in ?.
